# Patterns and causes of hospital maternal mortality in Tanzania: A 10-year retrospective analysis

**DOI:** 10.1371/journal.pone.0214807

**Published:** 2019-04-09

**Authors:** Veneranda M. Bwana, Susan F. Rumisha, Irene R. Mremi, Emanuel P. Lyimo, Leonard E. G. Mboera

**Affiliations:** 1 National Institute for Medical Research, Amani Research Centre, Muheza, Tanzania; 2 National Institute for Medical Research, Headquarters, Dar es Salaam, Tanzania; 3 SACIDS Foundation for One Health, Chuo Kikuu, Morogoro, Tanzania; Leibniz Institute for Prevention Research and Epidemiology BIPS, GERMANY

## Abstract

**Background:**

Maternal mortality is among the most important public health concerns in Sub-Saharan Africa. There is limited data on hospital-based maternal mortality in Tanzania. The objective of this study was to determine the causes and maternal mortality trends in public hospitals of Tanzania from 2006–2015.

**Methods and findings:**

This retrospective study was conducted between July and December 2016 and involved 34 public hospitals in Tanzania. Information on causes of deaths due to pregnancy and delivery complications among women of child-bearing age (15–49 years old) recorded for the period of 2006–2015 was extracted. Data sources included inpatient and death registers and International Classification of Disease (ICD)-10 report forms. Maternal deaths were classified based on case definition by ICD 10 and categorized as direct and indirect causes. A total of 40,052 deaths of women of child-bearing age were recorded. There were 1,987 maternal deaths representing 5·0% of deaths of all women aged 15–49 years. The median age-at-death was 27 years (interquartile range: 22, 33). The average age-at-death increased from 25 years in 2006 to 29 years in 2015. Two thirds (67.1%) of the deaths affected women aged 20–34 years old. The number of deaths associated with teenage pregnancy (15–19 years) declined significantly (p-value<0·001) from 17.8% in 2006–2010 to 11.1% in 2011–2015. The proportion of deaths among 30–34 and 35–39 years old (all together) increased from 13% in 2006–2010 to 15·3% in 2011–2015 (p-value = 0.081). Hospital-based maternal mortality ratio increased from 40.24 (2006) to 57.94/100000 births in 2015. Of the 1,987 deaths, 83.8% were due to direct causes and 16.2% were due to indirect causes. Major direct causes were eclampsia (34.0%), obstetric haemorrhage (24.6%) and maternal sepsis (16.7%). Anaemia (14.9%) and cardiovascular disorders (14.0%) were the main indirect causes. Causes of maternal deaths were highly related; being attributed to up to three direct causes (0.12%). Cardiovascular disorders and anaemia had strong linkage with haemorrhage. While there was a decline in the number of deaths due to eclampsia and abortion, those due to haemorrhage and cardiovascular disoders increased during the period.

**Conclusions:**

During the ten year period (2006–2015) there was an increase in the number of hospital maternal deaths in public hospitals in Tanzania. Maternal deaths accounted for 5% of all women of child-bearing age in-hospital mortalities. Most maternal deaths were due to direct causes including eclampsia, haemorrhage and sepsis. The findings of this study provide evidence for better planning and policy formulation for reproductive health programmes to reduce maternal deaths in Tanzania.

## Introduction

Childbirth is a natural process, though it has been associated with a number of risks, which may result into the death of either the baby, the mother or both. Globally, in 2015 maternal mortality ratio (MMR) was estimated at 216 per 100,000 live births [1]. Recent analysis of the global maternal mortality has indicated that 10·7 million women died due to maternal causes between 1990 and 2015; although there was an overall decreased trend by 43.9% during the period. Despite the overall decline in MMR since 1990, the ratio is 15 times higher in low-income than high-income countries [2]. The maternal mortality ratio in Sub-Saharan Africa stands at 546 per 100,000 live births, accounting for about two thirds of the global maternal deaths [1–2]. Tanzania is among the countries in Sub-Saharan Africa with highest MMR. The most recent population-based surveys indicate that the mean MMR in Tanzania is 556 per 100,000 live births [3] while the United Nations estimates put the figure at 950 per 100,000 live births [2]. For almost three decades (1990–2016) MMRs in Tanzania have remained high, with no sign of a significant reduction despite several efforts [4–6].

Maternal deaths are associated with both direct and indirect obstetric causes. The direct causes, which include haemorrhage, hypertensive disorders, obstructed labour, and sepsis are responsible for about three quarters of maternal deaths worldwide [7–10]. On the other hand, indirect causes of maternal death include the effects of pre-existing disorders, such as HIV, malaria, tuberculosis, mental diseases, epilepsy, and diabetes [8,11]. Several factors have been associated with maternal deaths; and they include antenatal care, maternal education [12], age and gravidity [13, 14]. For instance, the highest parity-specific maternal mortality ratios have been reported among the grand multiparous women [13]. Education enables access to information and helps empower women and their spouses to make appropriate and prompt decisions during pregnancy [15].

Generally, vital registration is considered to provide accurate and timely estimates of maternal mortality [15, 16]. However, studies have shown that in low-income countries the vital registration systems capture only a small fraction of deaths occurring in the community [17]. In Tanzania, most data on maternal mortality ratio are derived from population surveys [3, 18–19] and Population and Housing Census [20]; which are likely to be affected by recall ability of the respondents, and are available only after every five and 10 years, respectively. Hospital records are important sources of maternal deaths, as they are readily available and suffer less quality issues as compared to those from vital registration systems, can be used to monitor the patterns and causes needed for timely actions during care. Moreover, hospital deaths are certified by qualified health providers and hence can be used to identify areas that require improvement in maternal care provision.

There is limited utilization of data on hospital-based maternal mortality in Tanzania and other Sub-Saharan African countries despite the inclusion of maternal deaths in the national surveillance systems since 2004 [21]. By 2010, nearly, a third of all districts in sub-Saharan Africa had not integrated maternal mortality among the immediate notifiable events in their Integrated Disease Surveillance and Response programmes [22]. Furthermore, a review of the status of maternal mortality surveillance in 2012 showed that data on maternal deaths are lacking or incomplete in about half of the countries involved [23]. Weak health information systems in most low-income countries have resulted to very little attempts to analyse and use hospital-based data on maternal death which could provide local-specific evidence for appropriate planning and management. This study was therefore, carried out to determine the causes and maternal mortality trends in public hospitals of Tanzania from 2006 to 2015.

## Methods

### Study sites and sampling framework

The health care system in Tanzania include primary health facilities (dispensary and health centre), district hospital, regional referral hospital, zonal referral hospital and national hospital. Note that there is one regional referral hospital per region and one district hospital within a district. At the time of the study, there were 269 hospitals in Tanzania (public = 44.6%; private /faith-based organizations = 55.4%). The study involved 34 public owned hospitals from all levels. This is about one third of the public hospitals and 15% of all hospitals in the country. A sampling technique used to include study hospitals is described here after. First, the regions were categorised into three strata based on their proportional contribution to the national population. The strata were high populated regions (Dar es Salaam, Mwanza and Mbeya), medium populated regions (Kagera, Tabora, Morogoro, Kigoma, Dodoma and Tanga) and low populated regions (Arusha, Geita, Iringa, Katavi, Kilimanjaro, Lindi, Manyara, Mara, Mtwara, Njombe, Pwani, Rukwa, Ruvuma, Shinyanga, Singida and Simiyu) [20]. Second, the distribution of the hospitals within the country and regions; epidemiological burden and spatial variations of malaria and HIV/AIDS endemicity [3]; patterns of child mortality and human resource coverage were reviewed and taken into consideration to ensure representation. Based on the review, it was seem that to include three hospitals from each of the high populated region; two hospitals from each of the medium populated region and one hospital from each the low populated region will bring a reasonable representation. All the national, zonal referral and regional hospitals were purposely included in the study. In regions where the national or zonal referral hospital was included, the respective regional hospital was excluded. To obtain the needed number of hospitals for highly and medium populated regions, 10 district hospitals were included. These were randomly selected, for each region separately, excluding the district where the regional hospital was located. We assumed a homogeneous availability and quality of basic and comprehensive emergency obstetric care between hospitals hence not use that among sampling and inclusion criteria. The study setting and design has been described in details elsewhere [24].

### Source of mortality data

In Tanzania, the procedure used to collect information on causes of in-hospital death is standardized for all levels of hospital. Once an admitted patient dies, the physician on call will certify the death and then use the details of the case (written in the inpatient file), confirmed diagnosis, and complications that arises, to establish the sequence of events and determine the immediate, probable, and underlying cause of death. This information is filled in a death register in duplicate, of which a copy is retained at the respective hospital while the original form is submitted to the Registration Insolvency and Trusteeship Agency. As from 2013, this data is also filled in a death report form of which contents are entered in an electronic District Health Information System (DHIS2) at the end of each month.

### Data collection

Data were collected between July and December 2016 using customized paper-based collection tools. Hospital records were manually extracted (as recorded) from the identified paper sources. A team of two research scientists and four data collectors was working in one hospital at a time until all hospitals were covered. The research team and data collectors were trained on use of tools, including hospital registers and reporting forms, the types of data/variables required and ethical issues related to accessing medical records. Hospital staff, including the medical officer in-charge, a clinician, hospital matron/patron and members of the medical records unit, were oriented and involved to support the data collection exercise. Guided by hospital staff, a thorough search and compilation of all identified forms used to record mortality data was conducted. The extraction process started with the source with largest number of records, followed by others until all sources were assessed. Iteratively for each source, a date-to-date tracking was done to mark data completeness status. This process was repeated until all death events that occurred in the hospital were collected. Variables collected were the deceased’s age, sex, cause and date of death. This study utilized all records of causes of deaths due to pregnancy and delivery complications among women aged 15–49 years.

### Data analysis

Data were checked for mistakes and immediate errors before entered into Epi-Data database version 3·1. Data was later transferred to STATA Version 13 (StataCorp, College Station, TX, USA) for further processing and analysis. Maternal deaths were classified based on case definition by ICD-10 [25]. Age of the deceased women aged 15–49 years was categorized into a 5-year interval (15–19, 20–24, 25–29, 30–34, 35–39, 40–44 and 45–49 years). The aim was to study patterns of hospital-based maternal mortality by age, year and geographical zones. To do that various indicators were employed. First, proportions of overall age-specific hospital maternal deaths were calculated for the entire period (2006–2015), by collapsing data into 5-year periods (2006–2010 *versus* 2011–2015) and by specific years. The two periods, 2006–2010 and 2011–2015 were defined following the national targets for the 3.1 sustainable development goal which aimed that by 2030 countries should reduce maternal mortality ratio by at least two-thirds from 2010 baseline. The distributions of births and pregnant-related deaths obtained from the population survey (Source: www.nbs.go.tz - Mortality and Health Monograph of the Census, 2012) were compared with the hospital maternal deaths and total women hospital deaths obtained from our study. The aim was to detect any pattern that might be useful in strategies to reduce maternal deaths.

Two measures for maternal mortality i.e. maternal mortality ratio and the maternal mortality rate were picked and estimated at hospital level. The maternal mortality ratio is expressed per 100,000 live births and calculated and compared annually over the study period (2006–2015). The maternal mortality rate expressed per 1000 years of women exposure was calculated for 2004, 2010 and 2015 as reported in the national demographic surveys [3, 19, 26]. Similarly, the calculated maternal mortality ratio was overlaid with the crude birth rates (per 1,000 people) and the maternal mortality rate with deaths among all women for 2006 (reference for 2004 rates), 2009 and 2010 (reference for 2010 rates) and 2014 and 2015 (reference for 2015 rates) to detect useful trends and patterns. Population projections for 2006–2015 were also indicated for reference. Data on live births, crude birth rates and population were obtained from World Development Indicators databases (data.worldbank.org). To obtain the correct denominator to estimate hospital-based maternal mortality ratio, we adjusted the population live birth data by taking only the proportion of the institutional deliveries (i.e. facility-based births), which was conservatively taken as 50% of all births [3]. We then considered only births that occurred at hospitals which were assumed to be 40% of all health facility births [27]. Population statistics were used for this estimates as we couldn’t manage to obtain complete data on live births occurred in hospitals.

Causes of maternal deaths were categorized as direct and indirect causes. Conditions such as hypertension, heart attack, stroke, etc. were grouped as cardiovascular disorders. Indirect causes were considered only when a record of death was clearly specifying to occur while a woman was pregnant, at time of delivery or after child birth, or following abortion. Association between main causes of maternal deaths was studied and network plots were presented and discussed. Main causes of maternal deaths were ranked within each age category to guide actions and tailored interventions.

### Ethical considerations

This study received ethical approval from the Medical Research Coordinating Committee of the National Institute for Medical Research (Ref. No. NIMR/HQ/R.8a/Vol. IX/2230). Permissions to access hospital registers and reporting documents were sought from the Ministry of Health, Community Development, Gender, Elderly and Children and President’s Office Regional Administration and Local Government through the respective Regional Administrative Secretaries and Hospital Authorities. No individual identifiable information like names of the deceased were extracted from the sources provided, however, all entries were given identification numbers.

## Results

### Maternal mortality pattern

Thirty-four public hospitals were included in this study. Of these, four were tertiary level (national and zonal referral), 20 were regional referral and 10 were district hospitals. A total of 40,052 deaths of women of child-bearing age (15–49 years) were recorded during the period of January 2006 to December 2015. Among these, there were 1,987 maternal related deaths which represent 5.0% of all women of child-bearing age deaths. Deaths varied significantly between age groups. The median age at death was 27 years (interquartile range: 22, 33). The average age at death increased from 25 years in 2006 to 29 years in 2015. The proportion of teenage (15–19 years) maternal deaths was 13.60% (271/1,987) which was higher compared to 4.80% (95/1,987) among elderly aged women (40–49 years) (Table 1). Two thirds (67.1%) of the maternal deaths was reported in women aged 20–34 years old. However, most of the births were from women in the 20–29 years category (Table 1). Within age groups, the hospital-based statistics indicated that, 9.26% (271/2,927) of deaths of women 15–19 years old were due to maternal causes, and these percentages decreased with increasing age (8.4%, 7.3%, 4.9%. 3.9%, 1.4% and 0.5% for the 20–24, 25–29, 30–34, 35–39, 40–44 and 45–49 years, respectively).

**Table 1:** The distribution of number and percentage of hospital- and population-based maternal deaths and births by age category.

	Hospital-based statistics				Population-based statistics*			
Age category	Total women deaths	Maternal related deaths	% Total women deaths	% Maternal related deaths	Number of births	% Births	Maternal related deaths	% Maternal related deaths
15–19	2,927	271	7.31%	13.60%	166,488	10.40%	1,250	8.30%
20–24	4,963	417	12.39%	21.00%	426,485	26.63%	2,211	14.69%
25–29	7,061	515	17.63%	25.90%	399,821	24.97%	2,300	15.28%
30–34	8,103	402	20.23%	20.20%	298,876	18.66%	2,244	14.90%
35–39	7,331	287	18.30%	14.40%	194,146	12.12%	2,343	15.56%
40–44	5,409	76	13.50%	3.80%	83,241	5.20%	2,324	15.44%
45–49	4,258	19	10.63%	1.00%	32,356	2.02%	2,384	15.83%
**Total**	**40,052**	**1,987**	**100.0%**	**100.0%**	**1,601,413**	**100.0%**	**15,056**	**100.0%**

*Data from Population and Housing Census, 2012

**Fig 1:**
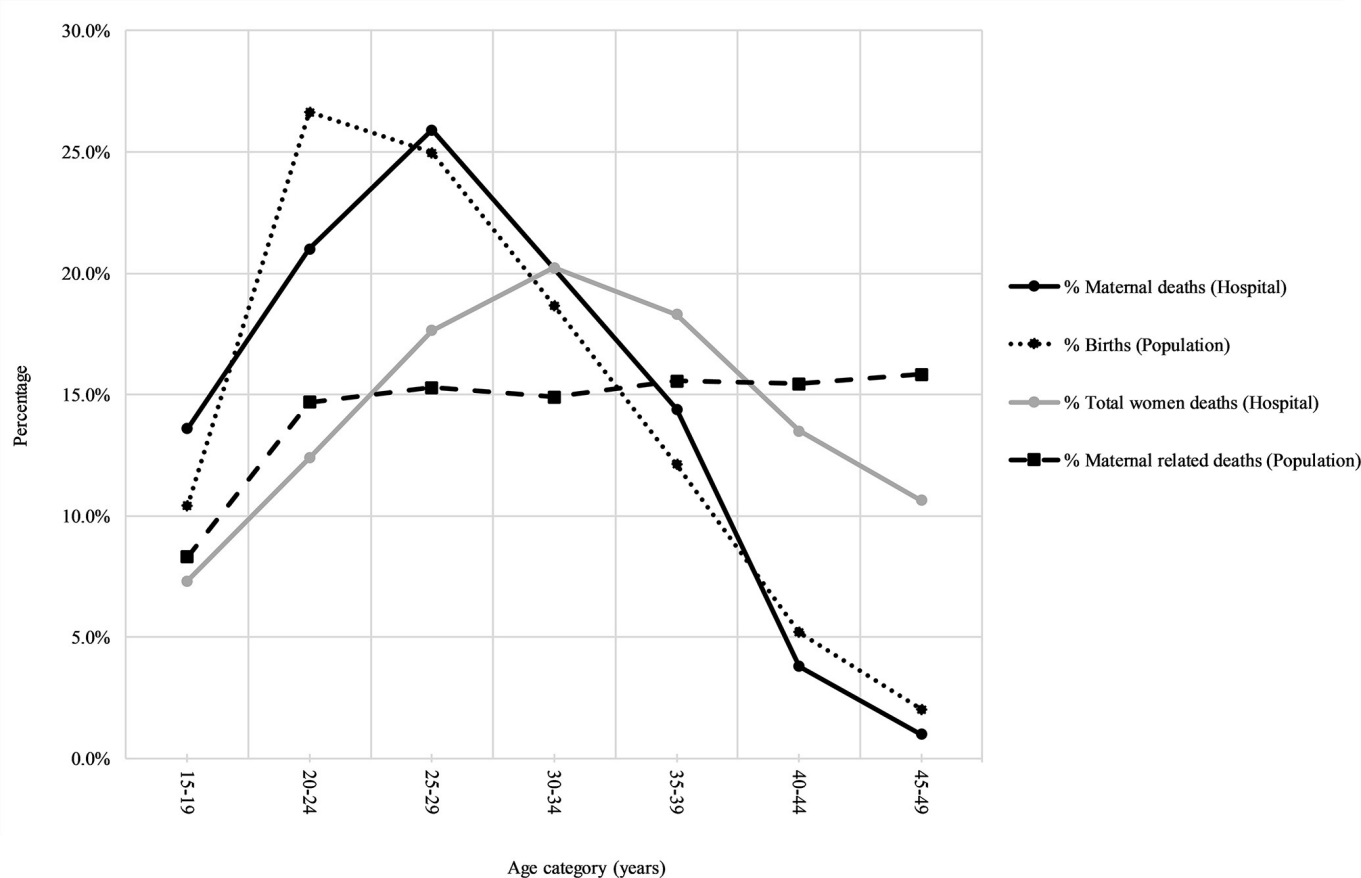
Percentage of birth, total women deaths and hospital maternal deaths by age category (Total women hospital deaths = 40,052; Hospital maternal deaths = 1,987).

The pattern of age-specific maternal deaths was similar to that of births which skewed at young aged women (Fig 1). During the 10-year period hospital maternal deaths peaked at the age group 25–29 years accounting for 25.9% of all maternal deaths and decreased sharply with age. On the other hand, the total women deaths peaked at 30–34 years (20.23%) and gradually decreased with increasing age. At population level statistics, births peaked at age of 20–24 years (26.63%), presented a slight decrease at 25–29 years (24.97%) then a sharp decline afterwards. Maternal related deaths presented a similar distribution from 20–24 years to 45–49 years (~15%). Maternal related deaths at population and hospital levels presented quite different age distribution patterns. Age distribution of population births and hospital maternal deaths presented a similar pattern from 25–29 years with proportions decreasing as women became older. However, there were significant differences at the 15–19 years and 20–24 years categories (Fig 1). Proportionally, more women (26.63%) were giving birth at 20–24 years of age but hospital data presented less mortality (21.00%) among this age group. A low proportion (10.40% of women aged 15–19 years were giving birth but had a relatively higher proportion of maternal deaths (13.60%) recorded (Fig 1).

**Fig 2:**
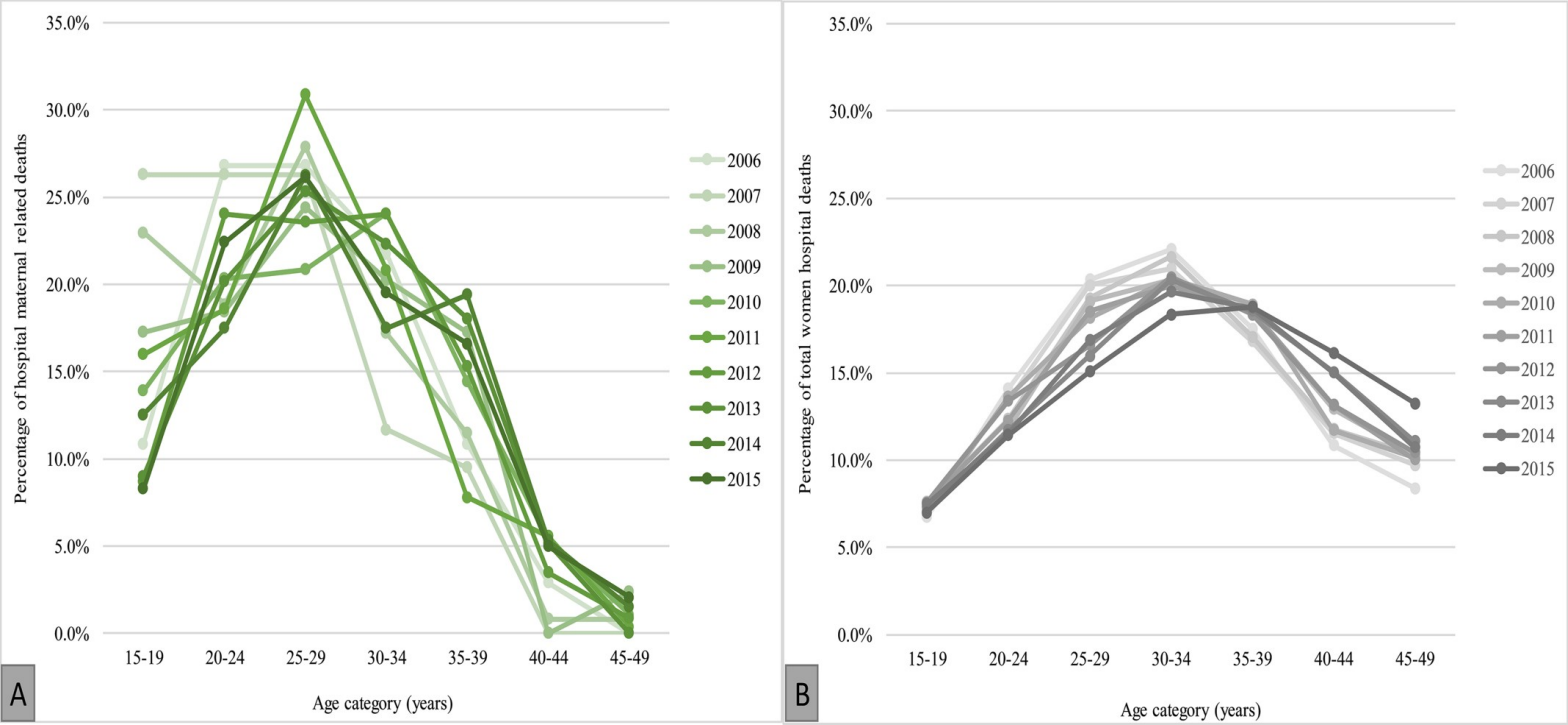
The percentage of hospital maternal associated deaths (A) and percentage of total women in-hospital deaths (B) by age category.

Annual trend of the proportions of age-specific maternal deaths parallel to that of women deaths are shown in Fig 2A and 2B. From 2013–2015 fewer number of young women died due to maternal causes (see dark green *versus* light green for age 15–19 years) while the proportion for middle-aged women slightly increased (see dark green *versus* light green for age >35 years). There were no significant changes among young women in the pattern of all women death. However, there was a reduction in proportion of mortality among middle-aged women and an increase in mortality among older women (Fig 2A and 2B).

**Fig 3:**
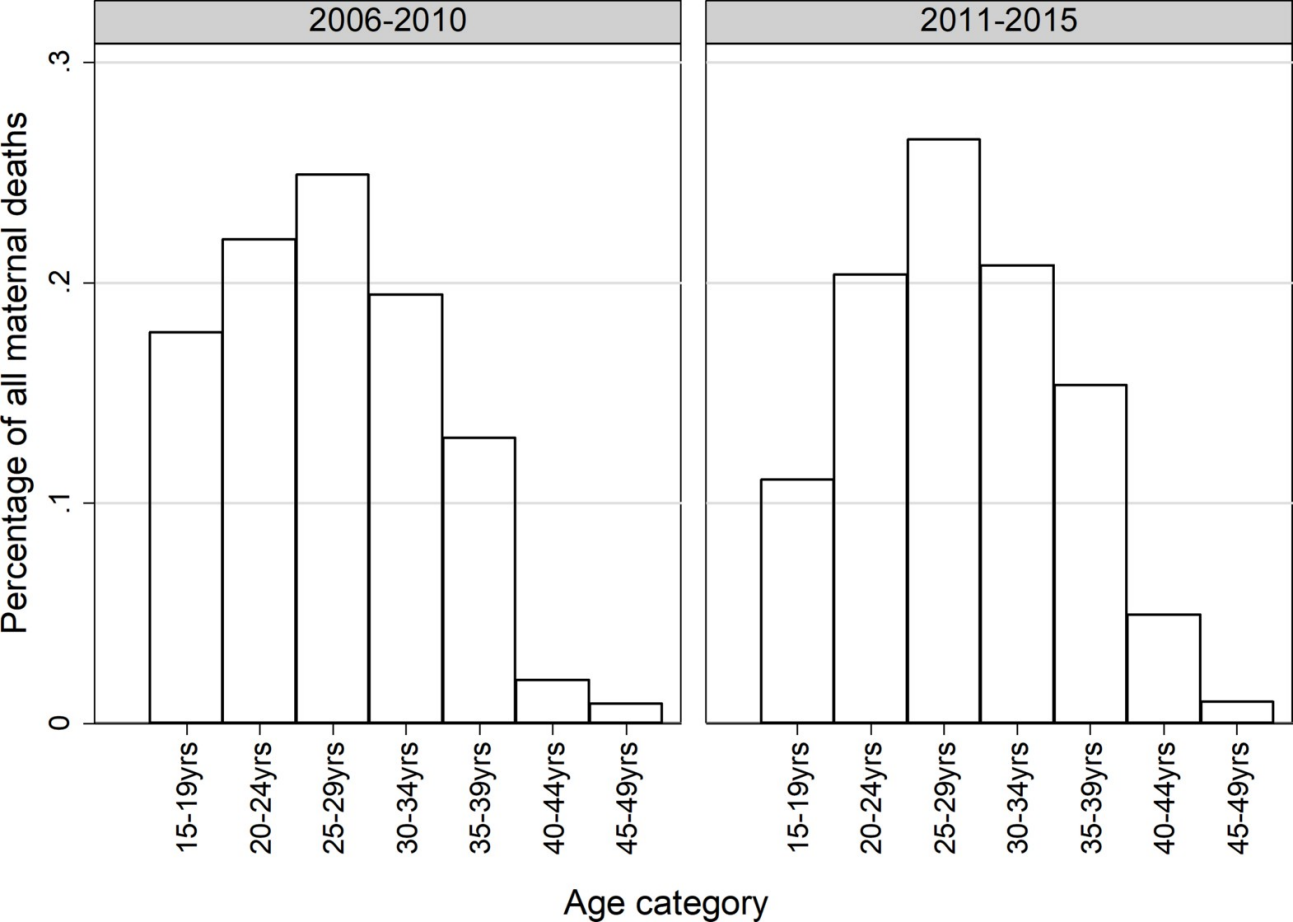
Proportion of all maternal related deaths by age comparison between 2006–2010 and 2011–2015 (2006–2010: n = 755; 2011–2015:n = 1,235).

Most maternal-associated deaths were reported among middle aged women in the age category of 30–44 years. These patterns indicate that as the young ones are slightly saved, maternal deaths push at mid aged and increase over time. Of the 1,987 deaths, 62·15% (n = 1,235) were reported during 2011–2015 while 37·85% (n = 755) during 2006–2010 period. Pulling the data together and comparing the two 5-year periods, the number of deaths due to early pregnancies (15–19 years) declined markedly from 17·8% during 2006–2010 to 11·1% during 2011–2015 (p-value <0·001, 2-sample proportional test). The proportion of deaths among the middle-aged women (30–34 and 35–39 years all together) categories increased from 13% to 15·3% (p-value = 0·081) (Fig 3).

### Maternal mortality ratio and maternal mortality rate

Estimated hospital-based maternal mortality ratio ranged from 33.65 to 69.64 per 100,000 births over the 10-year period under review (Table 2). There was an increase in maternal mortality ratio over the years, with the deaths per 100,000 live births increasing by over 40% over the 10-year period. On the other hand, the crude birth rate (CBR) declined from 42·2 per 1,000 people in 2006 to 36·8 per 1,000 in 2015. The largest number of maternal related deaths (n = 269) was recorded in 2011, during the same period of time when the highest maternal mortality ratio (69.64 per 100,000 births) was reported (Table 2).

**Table 2:** Population estimate, crude birth rate (CBR), live birth, maternal related deaths and hospital-based maternal mortality ratio (MMR) per year.

	Population-bases statistics^£^					Hospital-based statistics	
Year	Population estimate	CBR (per 1,000 people)	Live birth	Live births in health facilities (adjusted with 50%)	Live births in hospitals (adjusted with 40% of HF births)	Maternal related deaths	MMR (per 100,000 births)
**2006**	40,634,948	42.2	1,714,795	857,398	342,959	138	40.24
**2007**	41,923,715	42.1	1,764,988	882,494	352,998	137	38.81
**2008**	43,270,144	41.9	1,813,019	906,510	362,604	122	33.65
**2009**	44,664,231	41.5	1,853,566	926,783	370,713	168	45.32
**2010**	46,098,591	41.1	1,894,652	947,326	378,930	187	49.35
**2011**	47,570,902	40.6	1,931,379	965,690	386,276	269	69.64
**2012**	49,082,997	40.1	1,968,228	984,114	393,646	229	58.17
**2013**	50,636,595	39.6	2,005,209	1,002,605	401,042	233	58.10
**2014**	52,234,869	39.1	2,042,383	1,021,192	408,477	263	64.39
**2015**	53,879,957	38.6	2,079,766	1,039,883	415,953	241	57.94

^**£**^Source: data.worldbank.org

**Fig 4:**
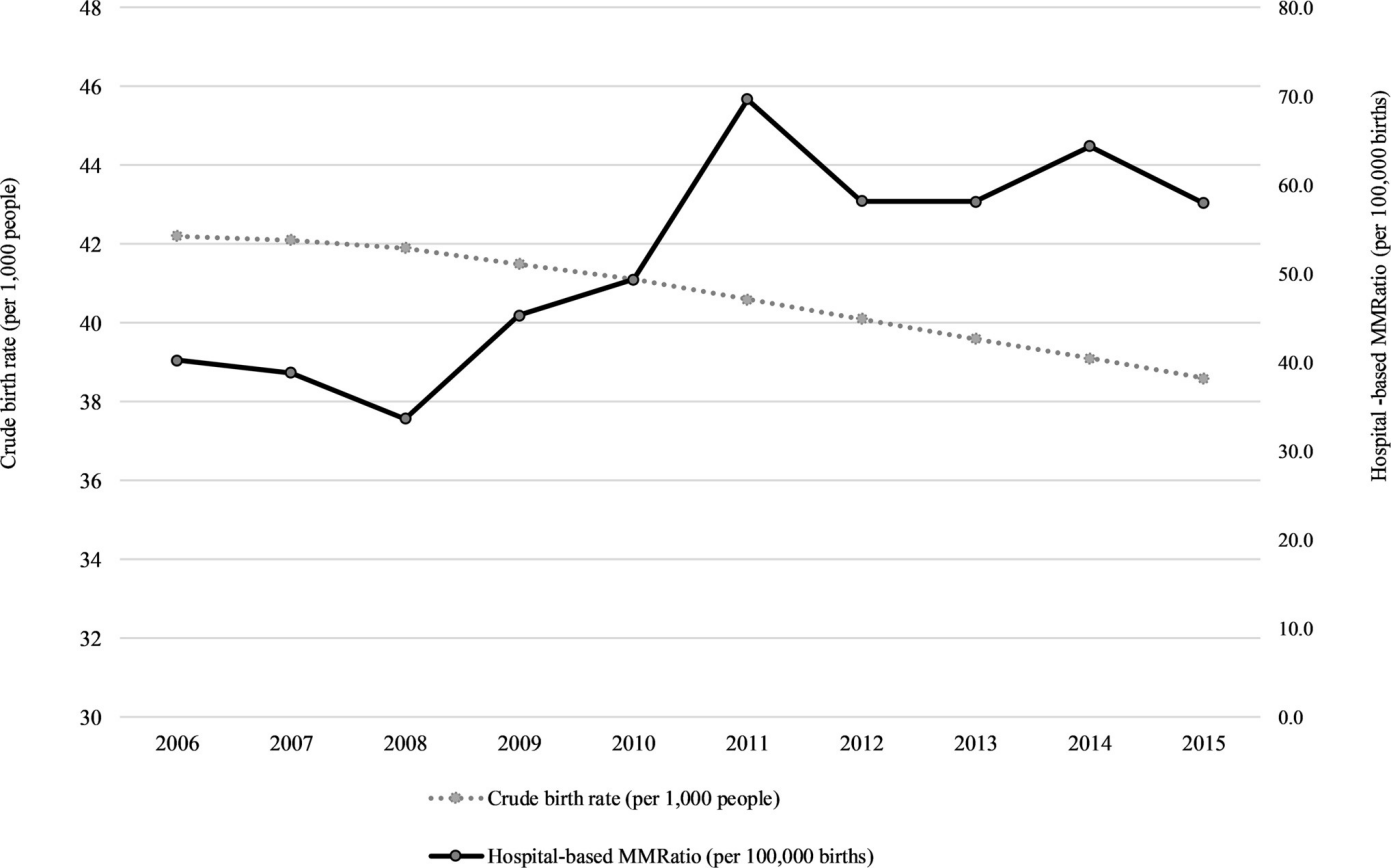
Annual crude birth rate per 1000 people and hospital-based MMR per 100,000.

The pattern of deaths showed a marked increase from 2008, peaked in 2011, dropped in 2012 and thereafter slightly increased in 2014 (Fig 4). For the 10 year period, 2008 represented a slightly lower number of deaths compared to all other years. While MMR was increasing, there was a decrease in the crude birth rate (Fig 4).

**Fig 5:**
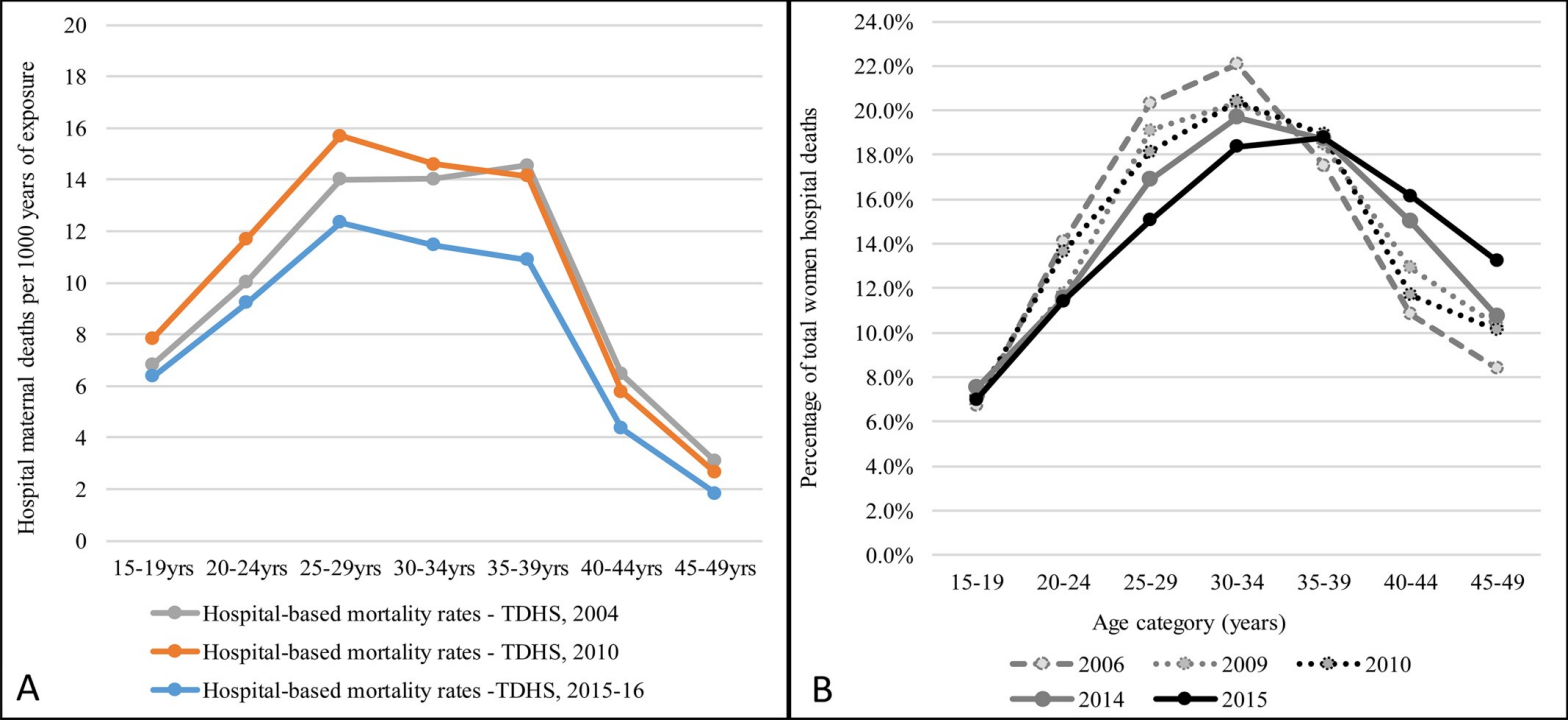
Age-specific hospital-based maternal mortality rates, 2006–2015.

Age-specific hospital-based maternal mortality rates using the years of exposures of 2004, 2010 and 2015 are presented in Fig 5. The rate was consistently low among young aged women then peaked for women aged 25–39 years. The maximum of 16 deaths per 1,000 women exposure years was observed among 25–39 years old women. The patterns for the 2004 and 2010 were quite comparable, however the rates were lower for 2015–2015 exposure. The pattern for proportion of all women deaths by age is presented along the mortality rates. The patterns indicate a strong correlation between general women mortality and maternal mortality rates.

### Causes of maternal mortality

**Fig 6:**
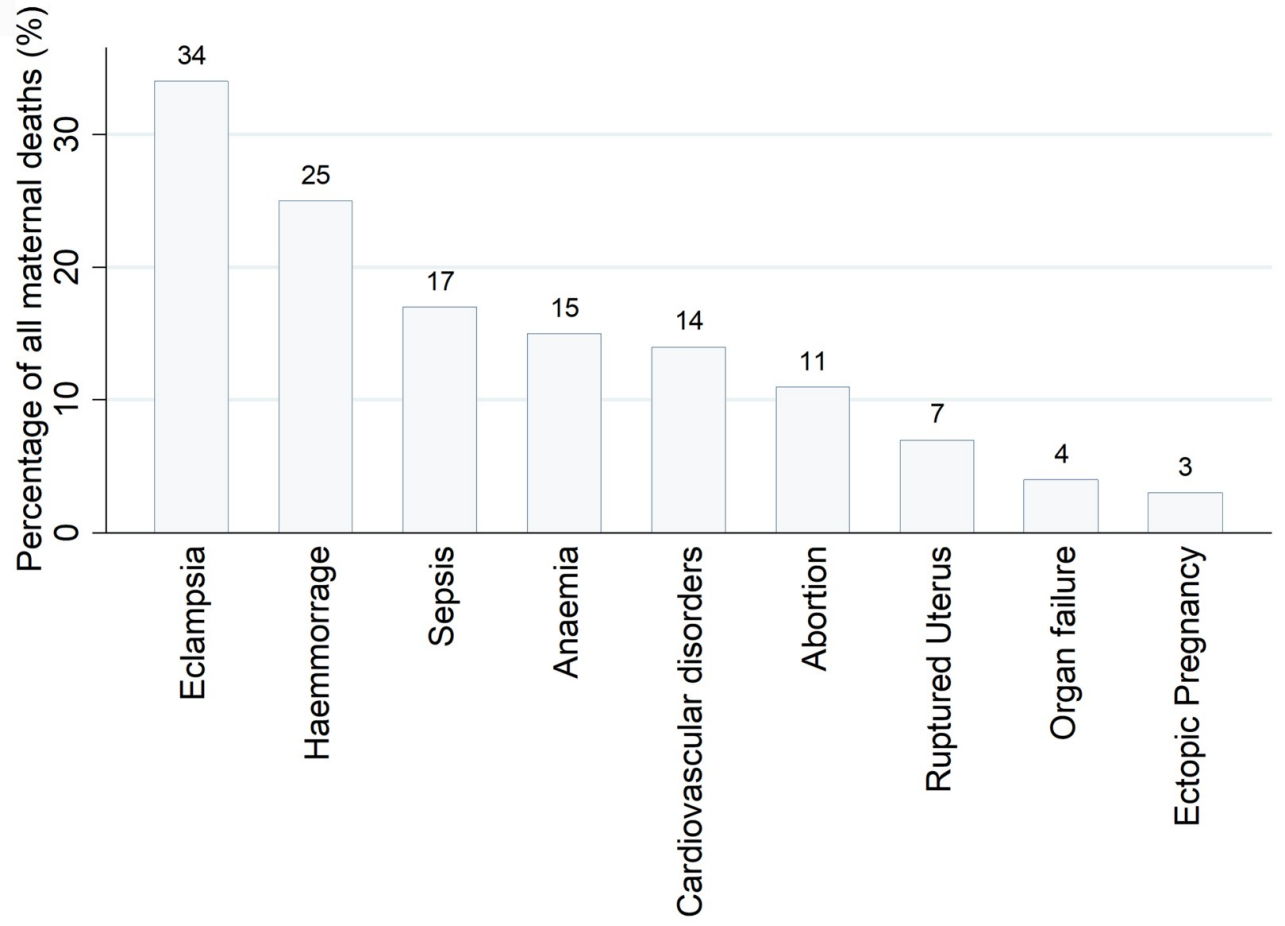
The proportion of major direct and indirect causes of maternal deaths, 2006–2015 (n = 1,987).

Of the 1,987 maternal deaths, 83·8% (n = 1,666) were due to direct causes and 16·2% (n = 321) were due to indirect causes. Major direct causes of maternal deaths were eclampsia (34%, n = 669), obstetric haemorrhage (24·6%, n = 488), maternal sepsis (16·7%, n = 336), abortion (10·8%, n = 215) and ruptured uterus (7·1%, n = 140) (Fig 6). Anaemia (14·9%, n = 295) and cardiovascular disorders (14·0%, n = 274) accounted for the highest percentage of the indirect causes, followed by malaria (1·3%), respiratory diseases (1·0%) and HIV/AIDS (0·8%). Other indirect causes were diabetes (0·4%), meningitis (0·35%) and tuberculosis (0·05%).

Eclampsia, haemorrhages, cardiovascular disorders and organ failure were the major causes of maternal deaths reported in zonal hospitals. The major causes of maternal deaths in the regional referral and district hospitals were haemorrhage, sepsis, anaemia, abortion, ruptured uterus and ectopic pregnancy.

An analysis was done to study common co-morbid direct causes that were reported to be associated with the primary cause of maternal death (Table 3). Maternal deaths were attributed to up to three direct causes (0·12%) and most frequently to two causes. The highest observed associations were between abortion and maternal sepsis (8·3%, n = 138) followed by ruptured uterus and haemorrhage (2·4%, n = 40) and eclampsia and haemorrhage (1·14%, n = 19). Maternal sepsis was associated with most of the main direct causes. Ectopic pregnancy and organ failure were the least co-morbid conditions reported (Table 3).

**Table 3:** Associated direct causes to the reported primary maternal cause of death (n = 1,666).

N	Percentage	Primary	Associated-1	Associated-2
138	8·28%	Abortion	Sepsis	-
40	2·40%	Ruptured uterus	Haemorrhage	-
19	1·14%	Eclampsia	Haemorrhage	-
13	0·78%	Ruptured uterus	Sepsis	-
8	0·48%	Eclampsia	Sepsis	-
7	0·42%	Abortion	Haemorrhage	-
3	0·18%	Ectopic pregnancy	Haemorrhage	-
2	0·12%	Haemorrhage	Sepsis	-
2	0·12%	Eclampsia	Ruptured uterus	-
1	0·06%	Eclampsia	Abortion	-
1	0·06%	Abortion	Ruptured uterus	Sepsis
1	0·06%	Ectopic pregnancy	Abortion	Sepsis

**Fig 7:**
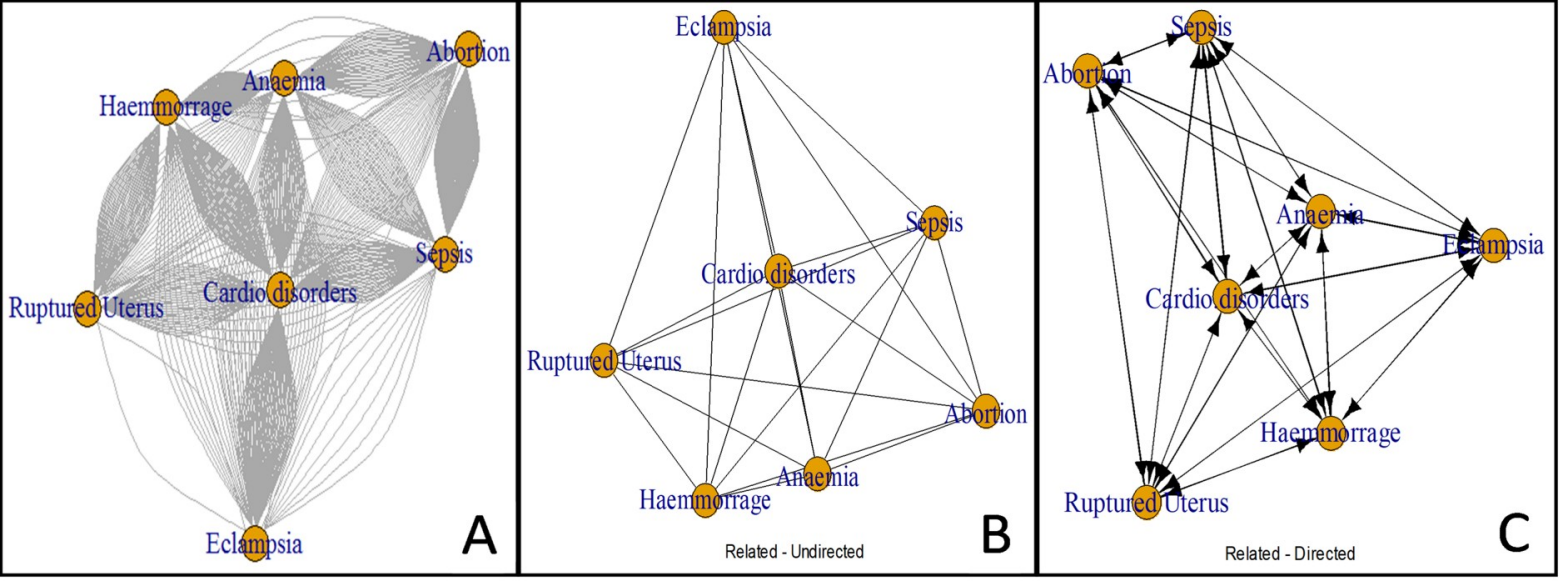
The relationship between main causes of maternal deaths indicating A) individual level network, B) related causes with no direction and C) related causes with direction.

To further analyse that, the network relationship (co-morbid) between main causes of maternal deaths is illustrated in Fig 7. The figure indicates the volume of individuals (Fig 7A), between causes without direction (Fig 7B) and with direction of the association (Fig 7C). It can be observed that cardiovascular disorders and anaemia were at the central point of the network with a strong linkage with haemorrhage. Ruptured uterus was strongly linked with haemorrhage, anaemia and cardiovascular disorders. Eclampsia was linked with cardiovascular disorders. Most deaths presented linkage between abortion, haemorrhage and sepsis. Arrows direction are in both ends indicating the multi-dimensional associations among all causes which complicates the understanding of chain of events.

Ranking the main causes of maternal deaths was done for each age category (Table 4). Eclampsia ranked as the number one killer among young women (15–34 years old). On the other hand, haemorrhage was leading cause of death among the older women aged 35–49 years (Table 4). Maternal sepsis and abortion ranked high among young women while ruptured uterus was more common among women older than 45 years.

**Table 4:** Ranking of the causes of maternal deaths by age category.

Age categories							
Rank	15-19yrs	20-24yrs	25-29yrs	30-34yrs	35-39yrs	40-44yrs	45-49yrs
**1**	**Eclampsia**	**Eclampsia**	**Eclampsia**	**Eclampsia**	Haemorrhage	Haemorrhage	Haemorrhage
**2**	Maternal Sepsis	Haemorrhage	Haemorrhage	Haemorrhage	**Eclampsia**	**Eclampsia**	Ruptured Uterus
**3**	Haemorrhage	Maternal Sepsis	Maternal Sepsis	Maternal Sepsis	Anaemia	Maternal Sepsis	Maternal Sepsis
**4**	Anaemia	Cardiovascular disorders	Anaemia	Anaemia	Cardiovascular disorders	Anaemia	Anaemia
**5**	Abortion	Anaemia	Cardiovascular disorders	Cardiovascular disorders	Maternal Sepsis	Cardiovascular disorders	Cardiovascular disorders
**6**	Cardiovascular disorders	Abortion	Abortion	Abortion	Ruptured Uterus	Ruptured Uterus	Ectopic pregnancy
**7**	Ruptured Uterus	Ruptured Uterus	Ruptured Uterus	Ruptured Uterus	Abortion	Abortion	**Eclampsia**
**8**	Organ failure	Organ failure	Organ failure	Organ failure	Organ failure	Organ failure	Organ failure
**9**	Ectopic pregnancy	Ectopic pregnancy	Ectopic pregnancy	Ectopic pregnancy	Ectopic pregnancy	Ectopic pregnancy	Abortion

**Fig 8:**
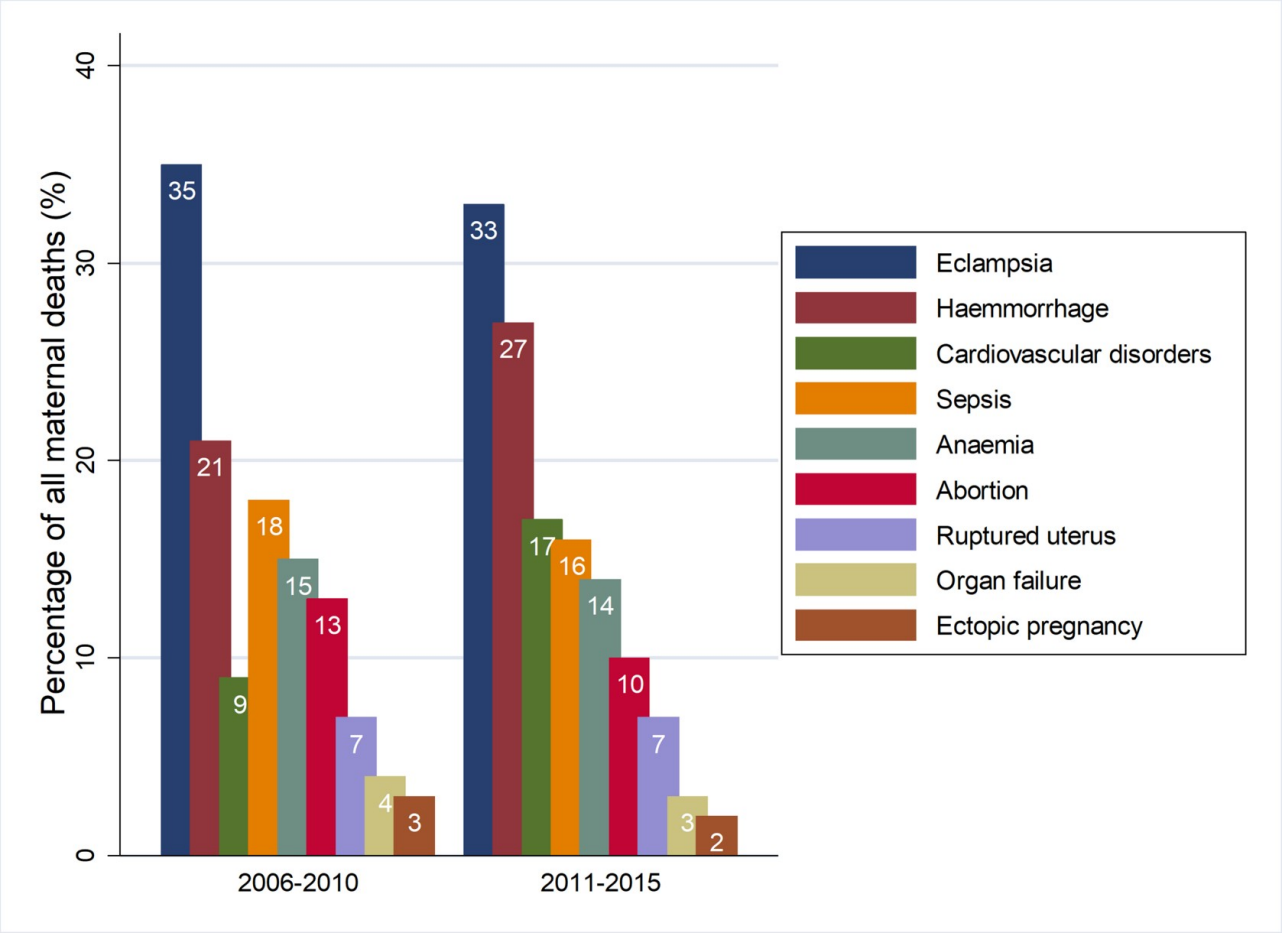
Comparison of the proportion of all major causes of maternal related deaths between the 2006–2010 and 2011–2015 periods (2006–2010: n = 755; 2011–2015:n = 1,235).

There was a slight decline in the proporton of deaths due to eclampsia (35% vs. 33%) and abortion (13% vs. 10%) between 2006–2010 and 2011–2015 periods. However, the proportion of deaths due to haemorrhage (21% vs. 27%) and cardiovascular disoders (9% vs. 14%) increased significantly (p<0·0001) from 2006–2010 to 2011–2015 period. The proportion of deaths due to ruptured uterus remained the same during the two 5-year periods (Fig 8).

**Fig 9:**
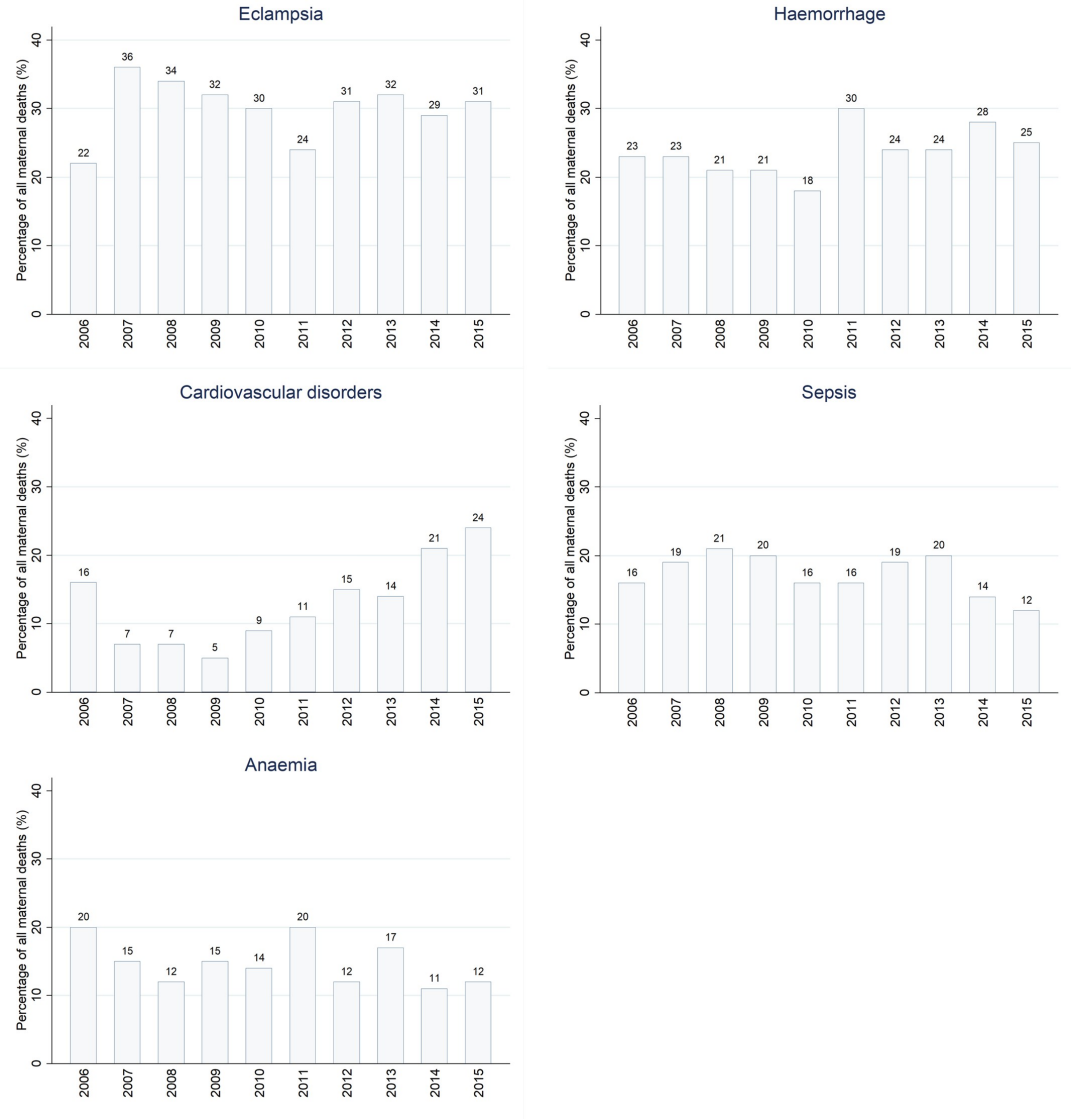
The trend of the major causes of maternal death over the 10 year period, 2006–2015.

Generally, a bimodal distribution in most of the major causes of maternal deaths was observed. From 2006 to 2010 the trend in mortality fluctuated between low and high proportions of causes of maternal deaths (Fig 9). The contribution of eclampsia to maternal deaths has remained constantly high except for a slight decline during 2011. Though there has been a slight decline in the contribution of haemorrhage to maternal deaths from 2006 to 2010, the mortality pattern started rising again during the period of 2011–2015. Maternal deaths attributed to cardiovascular disorders declined from 2006 to 2009 and started rising steadily from 2010. There were some indications in the decline of maternal deaths associated with sepsis during 2014–2015 (Fig 9).

### Maternal mortality by geographical region

**Fig 10:**
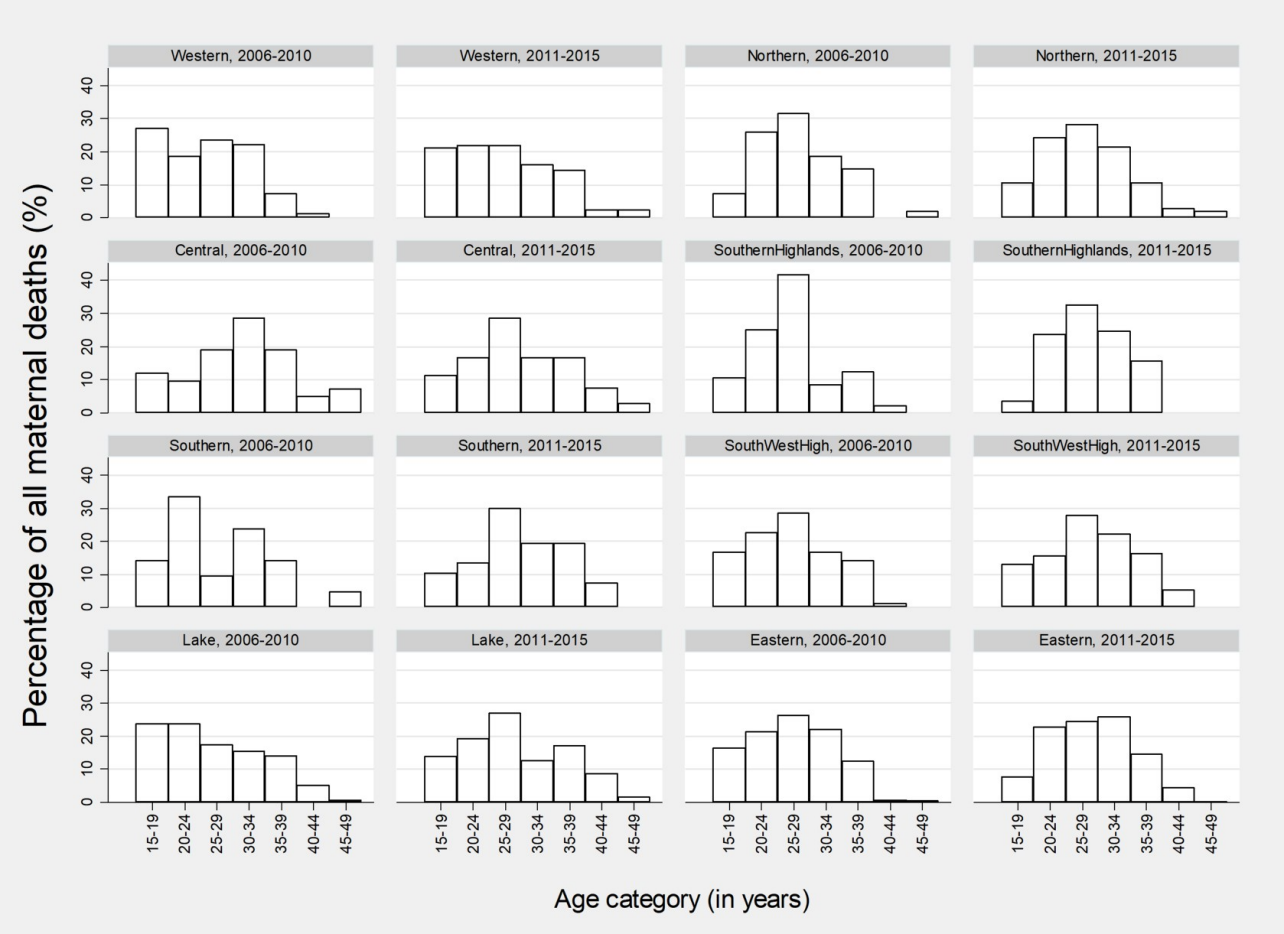
Percentage distribution for mortality patterns by age and geographical area comparing 2006–2010 and 2011–2015 (n = 1,987).

Maternal mortality varied between zone and regions of the country. Comparing five year periods, between 2006–2010 and 2011–2015, hospitals in the Western and Lake Victoria regions reported higher maternal mortality among the teenage age group (15–19 years) compared to other regions. The middle aged women died at a higher proportion in Southern highlands, South-western Highlands and Northern zones (Fig 10).

**Fig 11:**
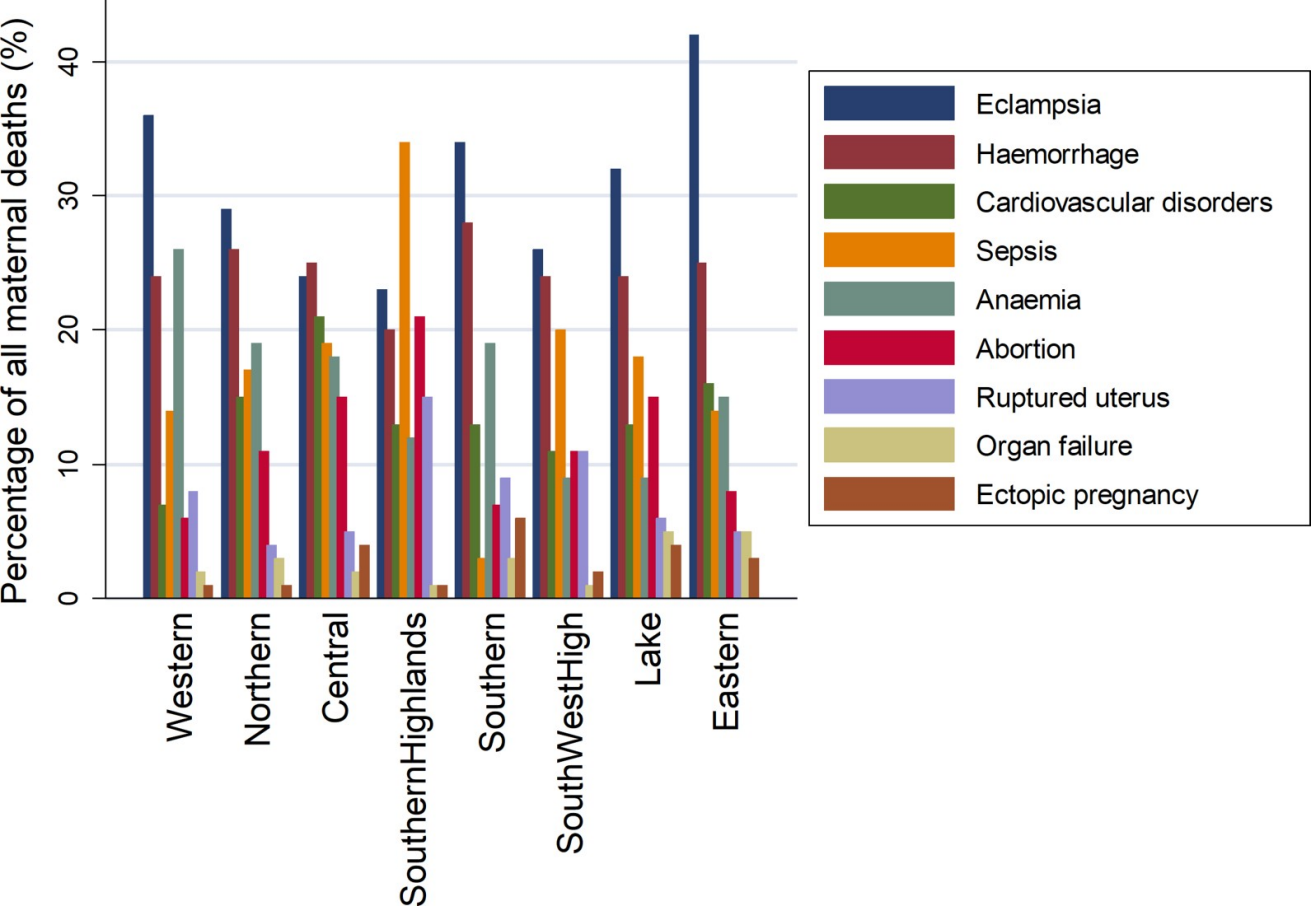
The distribution of major causes of maternal deaths by zone (n = 1,987).

During the 2006–2010 period most deaths in the southern zone were in the 20–24 years category. However, the age category shifted to 25–29 years in the second period of 2011–2015. Deaths among women over 45 years old were observed to decrease during the 2011–2015 period and this was clear for Southern, Southern Highlands and South-Western Highlands. Generally, the pattern was almost stable in Lake Victoria, Northern and Eastern zones (Fig 10). Deaths due to ruptured uterus were more prevalent in the southern highlands and south-western highlands. Deaths associated with abortion and sepsis were most prevalent in southern highland than in all other zones. Eastern, Lake Victoria and Western zones reported higher proportion of maternal deaths due to eclampsia (Fig 11).

## Discussion

This study utilized hospital statistics to understand the patterns and causes of maternal mortality in Tanzania. It should be understood that, even in place where mortality rates are high, maternal–related deaths are always rare events hence require attentive measures to be precisely estimated. A recent interest in understanding factors related to high maternal mortality in low-income countries, provokes many attempts to use all available and relevant data to study main causes, patterns, spatio-temporal trends and establish gaps that are useful in developing strategies to improve the situation at hand. This work is among the few that has estimated nation-wide hospital-based maternal mortality rates and maternal mortality ratios.

During the ten year period (2006–2015), the number of maternal deaths remained almost stable from 2006 to 2008 but increased gradually from 2009 to 2015. This pattern is supported by findings that the percentage of facility-based births has risen from 44% (1999) to 63% (2015) [3]. The majority of deaths were in the age category of 20–34 years. Deliveries occurring in facilities were also mostly among women 20–34 years old which could explain for the high proportion of deaths affecting this age category. The findings that maternal deaths affected the young women group have also been reported by other studies in specific hospitals in Tanzania [28–31] and other countries [31–35]. Several factors have been identified to be responsible for the high maternal deaths among young women. These include biological, economic, and cultural factors, malnutrition, immature reproductive tract, child marriage, and gender inequities [36–37]. It is important therefore that reproductive health programmes provide education, family planning services, and pre- and post-natal care services to reduce mortality among young women.

Most maternal deaths were attributed to direct obstetric causes mainly eclampsia, haemorrhage and sepsis. Similar findings have been reported by other studies elsewhere in Tanzania [28–29,31, 38], Kenya [12], Angola [39], Nigeria [13,40], Bangladesh [41] and Pakistan [42]. Globally, eclampsia is one of the leading causes of maternal mortality [43]. Although, there has been a significant reduction in the rates of eclampsia in high-income countries, it has remained high in the low-income countries [43]. In this study, eclampsia ranked number one killer among young women while haemorrhage was the number one killer among the older women. Similar to our findings, higher prevalence of eclampsia among adolescents and young women has been reported in other studies in Asia and other regions [44–46]. In contrary, a study in Taiwan has indicated that the incidence of pre-eclampsia was the lowest in the 20–24-year age group and higher among >35-year age groups [47]. Preeclampsia and eclampsia are more frequent among women in their first pregnancy, women who are obese, women with pre-existing hypertension, and those with diabetes [48]. The higher direct obstetric causes of maternal death in our study was similar to those reported in other low-and middle income countries [11]. Statistics indicate that about two-thirds of maternal deaths in Africa are related to direct obstetric complications mainly haemorrhage, hypertension, sepsis, and obstructed labour [8]. In a study in a rural district of Tanzania, the major causes of death were postpartum haemorrhage and obstructed labour [49]. Several studies point out to consistently elevated risk of maternal mortality from ruptured uterus [50–53]. Generally in low-income countries, most cases due to ruptured uterus are due to ignorance, poverty, unavailability of skilled staff and poor supply of essential medical supplies [54–56].

In our study the major indirect causes were attributed to anaemia, cardiovascular disorders, malaria, HIV/AIDS and meningitis. Similar indirect causes have been reported by other studies in Tanzania [29,35,38] and other low and middle-income countries [8,13,57]. Statistics indicate that globally, 27·5% of all maternal deaths results from indirect causes, with the highest proportions in Sub-Saharan Africa and South Asia [8,10]. Despite this contribution to indirect causes, they have received little attention as most national and international efforts are directed toward direct causes of maternal deaths–focusing on emergency obstetric care [48,58–59]. It is important that studies to understand these indirect causes of death are carried out to provide evidence to develop appropriate interventions to holistically reduce maternal mortality.

There was a network relationship between main causes of maternal deaths. For instance, cardiovascular disorders and anaemia were at the central point of the network with a strong linkage with haemorrhage. Ruptured uterus was strongly linked with haemorrhage, anaemia and cardiovascular disorders. Most deaths presented linkage between sepsis and abortion. The associations observed indicate that all these causes are related to each, which sometimes pose difficulties in management. In a number of countries, haemorrhage has been reported as the most consistently important cause of death in hospital studies [32]. Haemorrhage has been associated with several conditions, including obstructed labour [60–61]. The association of anaemia and maternal mortality observed in this study is most likely to be a co-factor in death from haemorrhage [32] or nutritional deficiencies; usually lack of iron or folic acid [3]. Several studies have reported that postpartum haemorrhage is associated with anaemia [62]. Anaemia is most prevalent in Tanzania, with recent statistics indicating that 45% of women are anaemic [3]. To address post-partum haemorrhage in Tanzania, during the early 2010s, there have been efforts to improve active management of the third stage labour with emphasis on training and use of uterotonics [63]. However, a recent study has shown remarkable improvement in the quality of post-partum haemorrhage prevention at lower health facilities but not in hospitals [64]; emphasising the need to continue with efforts to improve the quality of care in hospitals including improvement in antenatal care [12].

There were variations in the maternal mortality ratio and causes by geographical regions and by hospital. The Southern highlands and South-western zones had a large number of maternal deaths due to ruptured uterus. There were higher proportions of maternal deaths associated with eclampsia in zonal referral hospitals than in the regional or district hospitals. This is likely to be associated with delays in referrals, with severe and complicated cases received late at zonal referral hospitals of which have low survival chances. Studies in Tanzania indicate that more than a quarter of maternal deaths are attributed to late referral from lower to higher care facilities, long distance to facility and poor infrastructure [31,50]. In practice the long distance to a health facility, poor communication infrastructure and transportation continue to complicate a timely access to health care due to delays [65–69]. In addition, this suggest that there is a weak health care system which contributes to poor management of these conditions at regional and district level hospitals. In addition, most healthcare facilities in low- and middle-income countries are unable to offer safe and effective care to women with obstetric complications due to limited resources [38, 70].

The reported high number of maternal deaths in this study could be attributed to a number of factors, both institutional and individual. Substandard care factors including patient and medical service [38], inadequate or lack of blood for transfusion, delay in receiving treatment and mismanagement have been described as among the most common factors [71]. Other medical factors include the delay in diagnosis and receiving treatment, as well as inadequate supplies or equipment needed for blood transfusion [31]. The lack of essential equipment, adequate number of competent staff and stock-out of essential drugs have also been reported as causes for delay in receiving timely and effective obstetric care [70–72]. A delay in the necessary referrals between healthcare facilities has also been reported as contributing factor to maternal deaths [70, 73]. Individual factors include culture and socio-economic status. Poverty and inequity have been described to undermine the survival of mother during pregnancy and after delivery [74]. Though maternal care in Tanzania is provided free of charge, substantial out-of-pocket payments are common [75–76]. Gender discrimination, low levels of female education, and inability to access care have been described to result into delays and unnecessary maternal deaths [77].

The hospital-based maternal mortality ratio was observed to increase substantially over the 10 years period. The ratio was 40/100,000 live births in 2006, peaked during 2011 (69/100,000 live births) and ~58/100,000 live births in 2015. These estimates are comparable to other studies done in other low-income countries [78], however much higher than those in high-income countries [79]. A hospital-based study conducted in Ghana covering a period of 1987–2000 reported a maternal mortality ratio of 1077/100,000 live births [80]; while a 3-year (2012–2014) tertiary hospital mortality ratio of 410/100,000 was reported in India [81]. Comparing the estimate obtained for same period from this study, the ratio is estimated at 60/100,000 which is much lower. These variations are due to the fact that our study included various levels of hospitals (district to tertiary hospitals). Most tertiary care hospitals receive referral cases from lower facilities, including other hospitals which are complex and at high risk.

This study has some limitations. The information on the causes of death was collected from hospital and was retrospective in nature. We relied on the available documented data from hospital registers and report forms which could be prone to misclassification or misreporting of the causes of death depending on who certifies the death. Moreover, hospital-based data provide estimates of maternal mortality that reflect the experience of a proportion of the population that seek hospital care during delivery. However, the findings of this study highlight the pattern, trend and causes of maternal deaths, information which is crucial for planning improvement in hospital management. Although the maternal mortality statistics from hospital-based studies are likely to be biased, under-reported and might not give the true picture of what is happening in the community, they complement estimates from population-based studies, and are important for examination of the causal mechanisms involved in mortality [32].

During the ten year period (2006–2015) there was an increase in the number of hospital maternal deaths in the public hospitals in Tanzania. Most maternal deaths were mainly due to eclampsia, haemorrhage and sepsis. This suggest that there are some deficiencies to recognise and manage obstetric complications which need attention. Results of this study demonstrate that even from the health care delivery point of view, Tanzania is still far from reaching the global targets for maternal health. Results from this study have set a foundation on the current state which could be used to develop tailored strategies that target reducing deaths that occur in hospital settings. This emphasise the need for reproductive health programme to not only focus on the physical availability of health care facilities, but also the quality of maternal care provided by these facilities including timely provision of emergence care. Further studies are necessary to clarify and expand the findings of this study which could explain the contributing factors associated with hospital maternal deaths.
